# Supplementary material for: Ultrafast fluxional exchange dynamics in electrolyte solvation sheath of lithium ion battery
Source: Nat Commun. 2017 Mar 8;8:14658. doi: 10.1038/ncomms14658 (PMC5344975; doi:10.1038/ncomms14658)
Supplement: Supplementary Information — Supplementary Figures 1-12, Supplementary Tables 1-2, Supplementary Notes 1-6 and Supplementary References [file ncomms14658-s1.pdf]

Supplementary Information

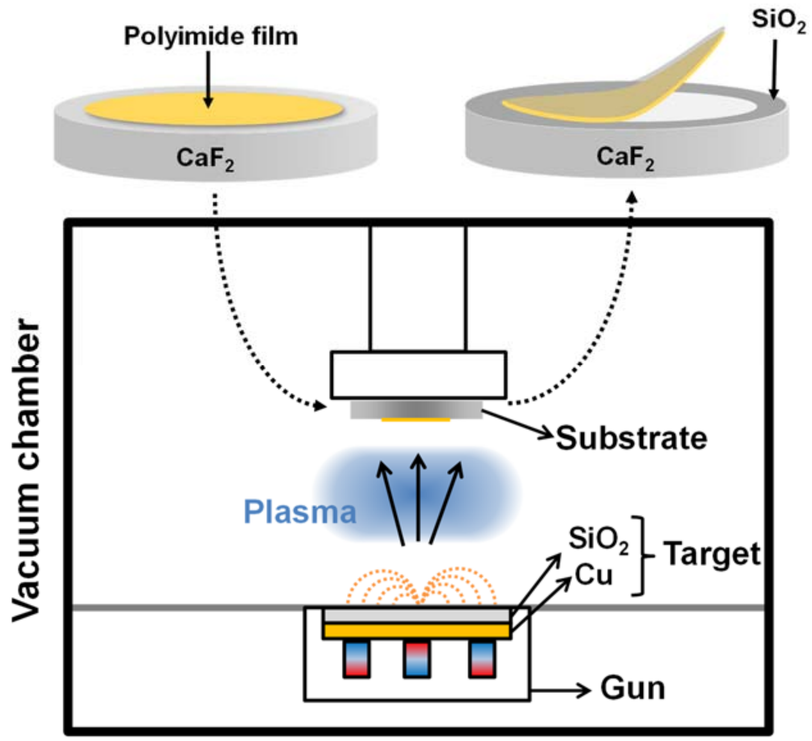

Supplementary Figure 1. Schematic diagram of the RF magneton sputtering system used to deposit a thin SiO_2 film on CaF_2 surface.

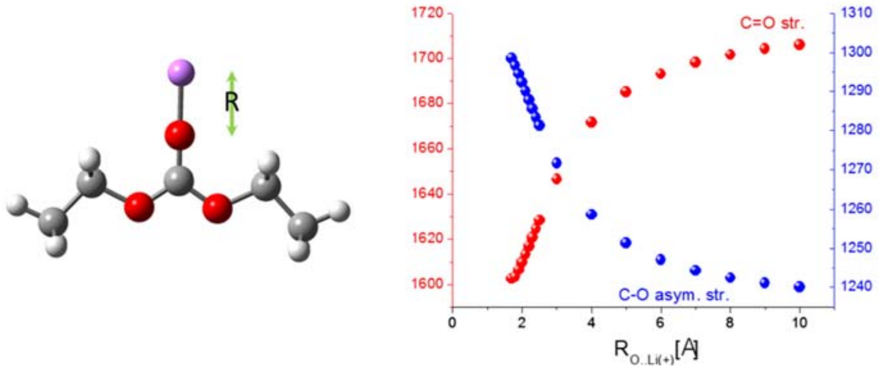

Supplementary Figure 2. A $Li^{+}\cdots$DEC complex structure. As the inter-atomic distance (R) between Li^{+} and carbonyl oxygen atom of DEC increases, the C=O stretch frequency increases (blue-shifts), whereas the O-C-O asymmetric stretch frequency decreases (red-shifts). These patterns are consistent with the FTIR data shown in Fig. 1.

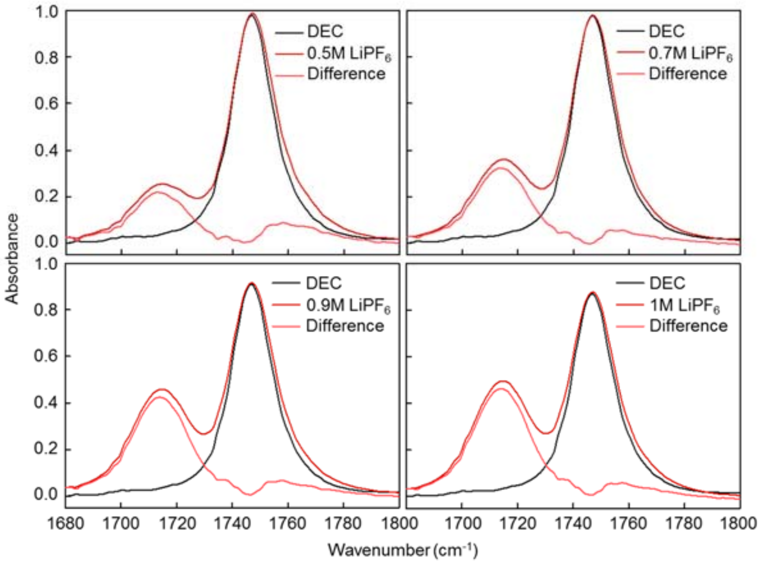

Supplementary Figure 3. Normalized C=O stretch IR spectra (blue line) of $LiPF_6$ DEC solutions at different concentrations. In each panel, the normalized C=O stretch IR spectrum of DEC in pure DEC liquid is also plotted for the sake of direct comparison. The difference spectrum between the two (blue and black lines) is shown in red.

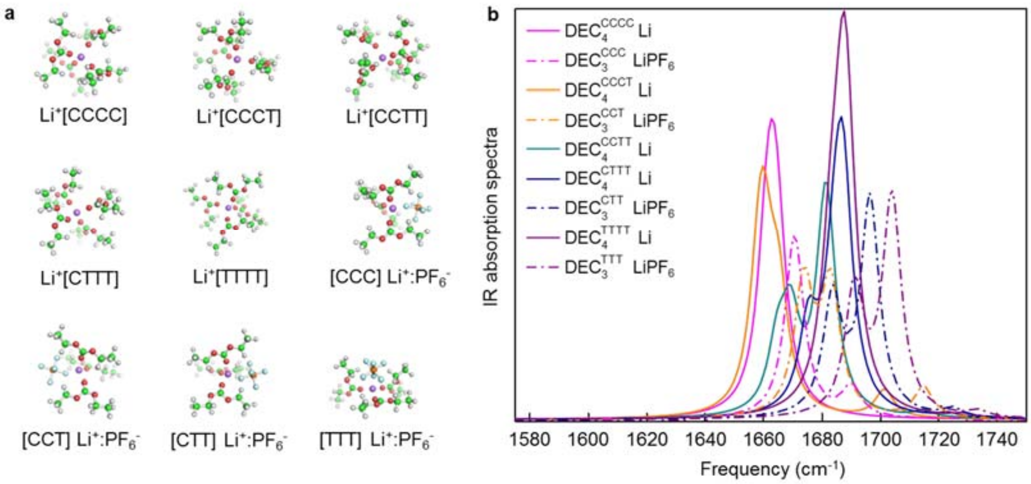

Supplementary Figure 4. Quantum chemistry calculation (a) Optimized structures of $(DEC)_4Li^+$ and $(DEC)_3$ Li^+:PF_6^- with different conformations of ethyl group of DEC. (b) The calculated IR spectrum with optimized conformer structures of $(DEC)_4Li^+$ and $(DEC)_3$ Li^+:PF_6^-. Here, the Lorentzian line width is assumed to be 8 cm^{-1}.

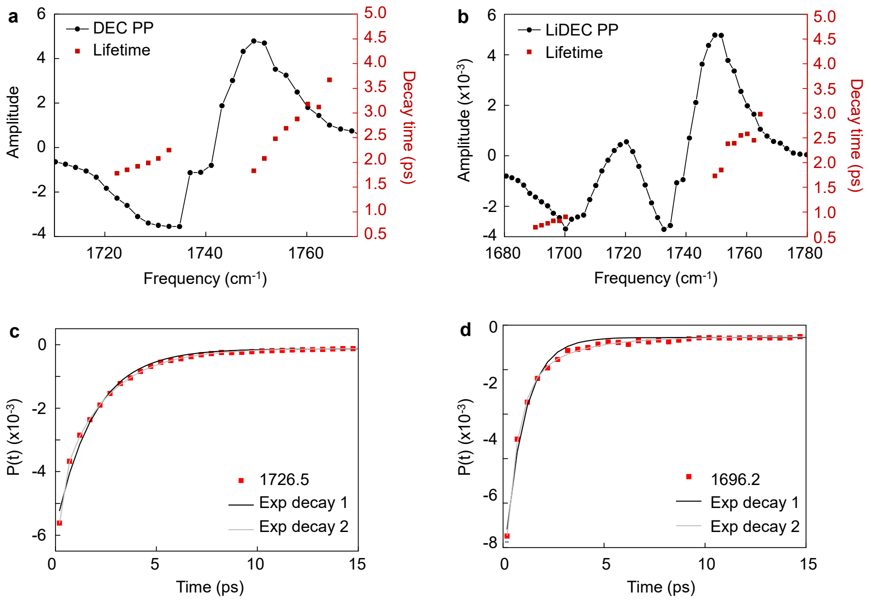

Supplementary Figure 5. Vibrational lifetime analysis results with the isotropic pump-probe data. In panels **a** and **b**, the IR pump-probe spectra of pure DEC liquid and 1.0 M $LiPF_6$ DEC solution, respectively, at waiting time of 0.3 ps are shown. In addition, the fitted exponential decay constants (red squares) at various probe frequencies are also plotted in the same panels. Representative exponential fitting results of isotropic pump-probe signals are shown in panels **c** and **d**.

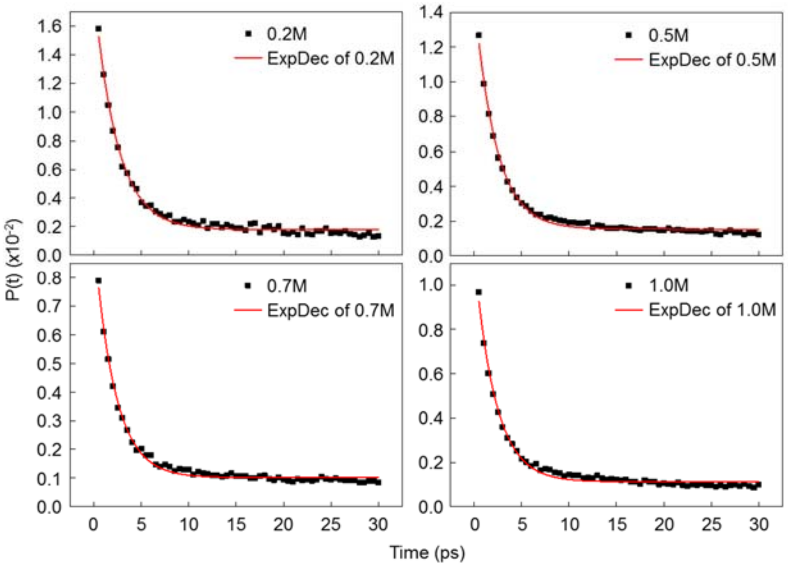

Supplementary Figure 6. Isotropic pump-probe signals of $LiPF_6$ DEC solutions at four different $LiPF_6$ concentrations. Red line is the single exponential fit.

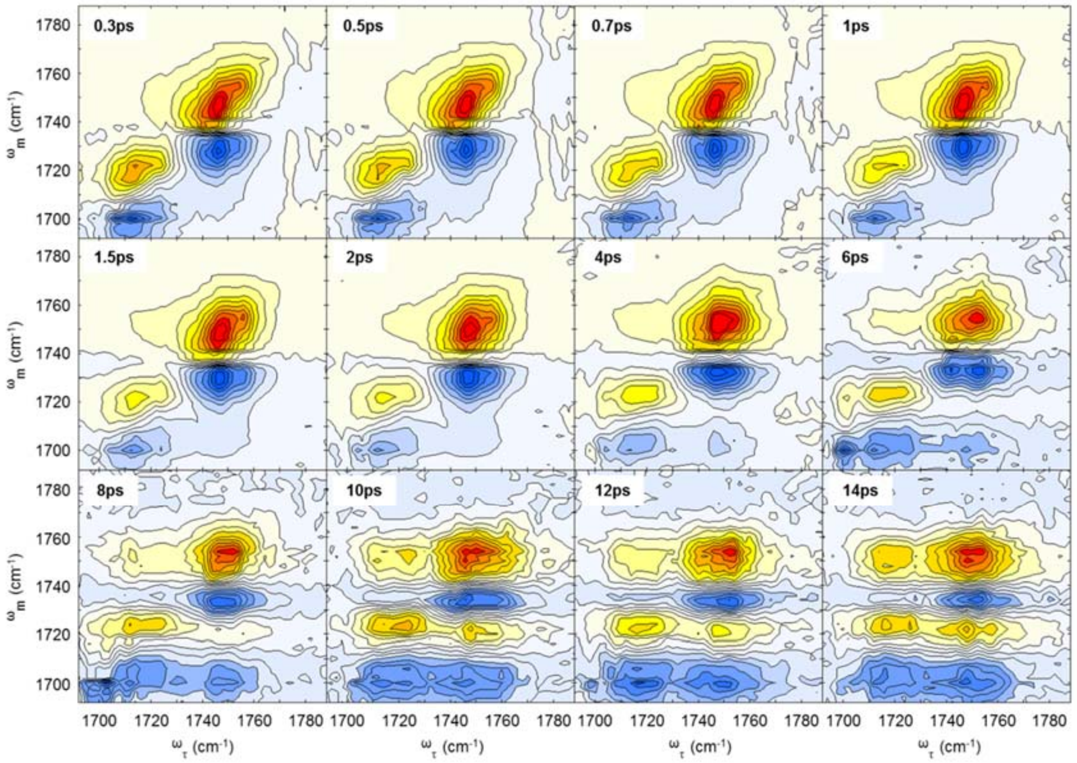

Supplementary Figure 7. Time-resolved C=O stretch 2DIR spectra of 1.0 M $LiPF_6$ DEC solution. The waiting times are 0.3, 0.5, 0.7, 1.0, 1.5, 2.0, 4.0, 6.0, 8.0, 10, 12, and 14 ps, respectively.

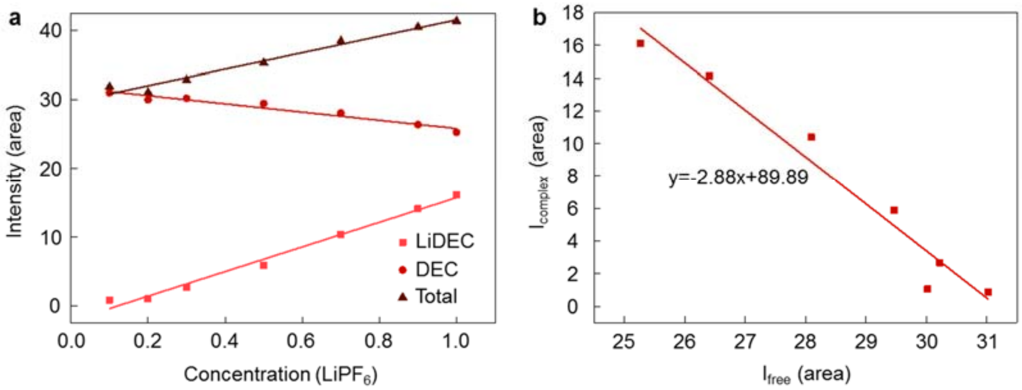

Supplementary Figure 8. Gaussian functions to fit the C=O stretch IR bands of free DEC and Li-DEC complex. (a) IR intensities (integrated areas) of free DEC (red), Li-DEC (orange), and total C=O stretch bands. (b) The intensity (integrated area) of Li-DEC complex is plotted with respect to that of free DEC.

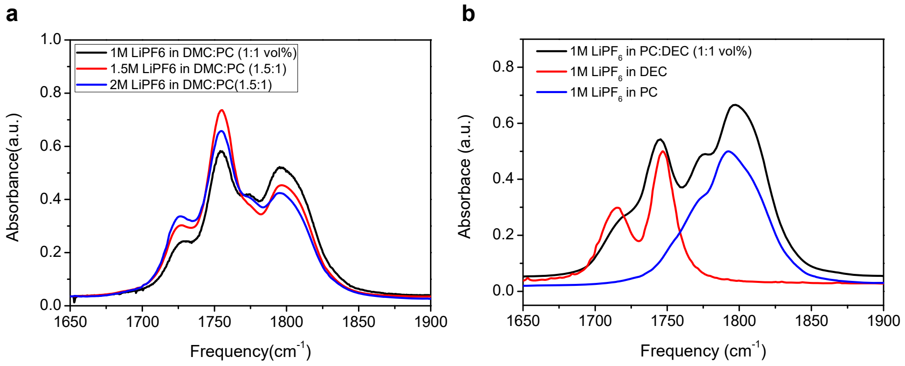

Supplementary Figure 9. Carbonyl stretch IR spectra of (i) 1.0 M $LiPF_6$ in DMC:PC (=1:1 in volume percent) solution, (ii) 1.5 M $LiPF_6$ in DMC:PC (= 1.5:1) solution, and (iii) 2.0 M $LiPF_6$ in DMC:PC (= 1.5:1) solution (a). The C=O stretch IR spectrum (black line) of 1.0 M $LiPF_6$ in DEC:PC (=1:1) solution is plotted in (b), where the red and blue lines correspond to the IR spectra of 1.0 M $LiPF_6$ in pure DEC solvent and 1.0 M $LiPF_6$ in pure PC solvent, respectively.

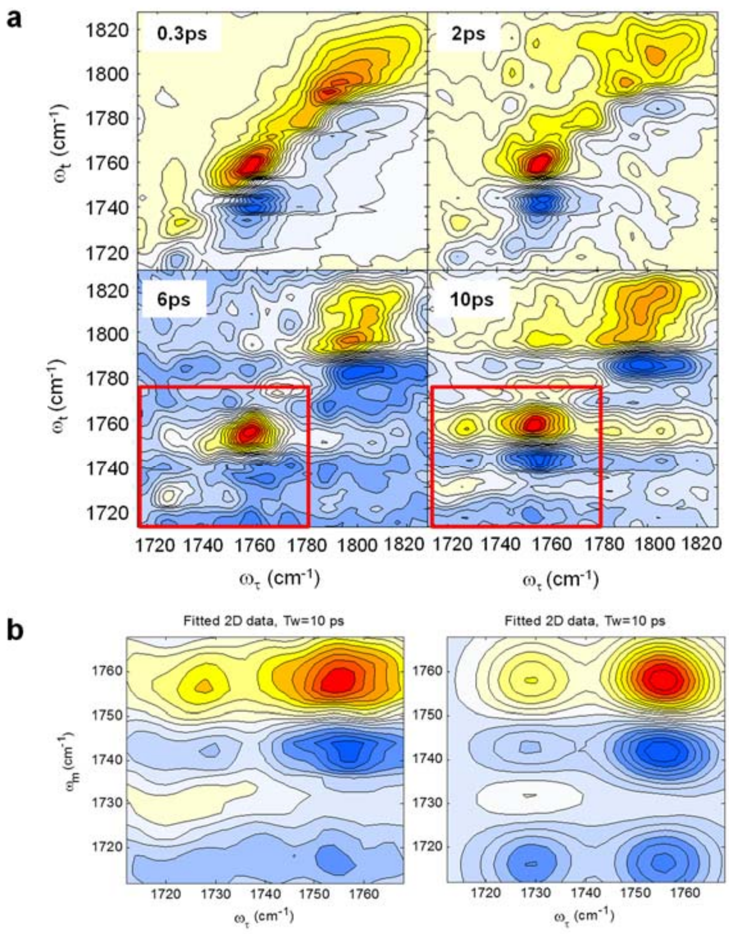

Supplementary Figure 10. Time-resolved 2DIR chemical exchange spectra for the solution (i), 1.0 M $LiPF_6$ in DMC:PC (=1:1 in volume percent) solution.

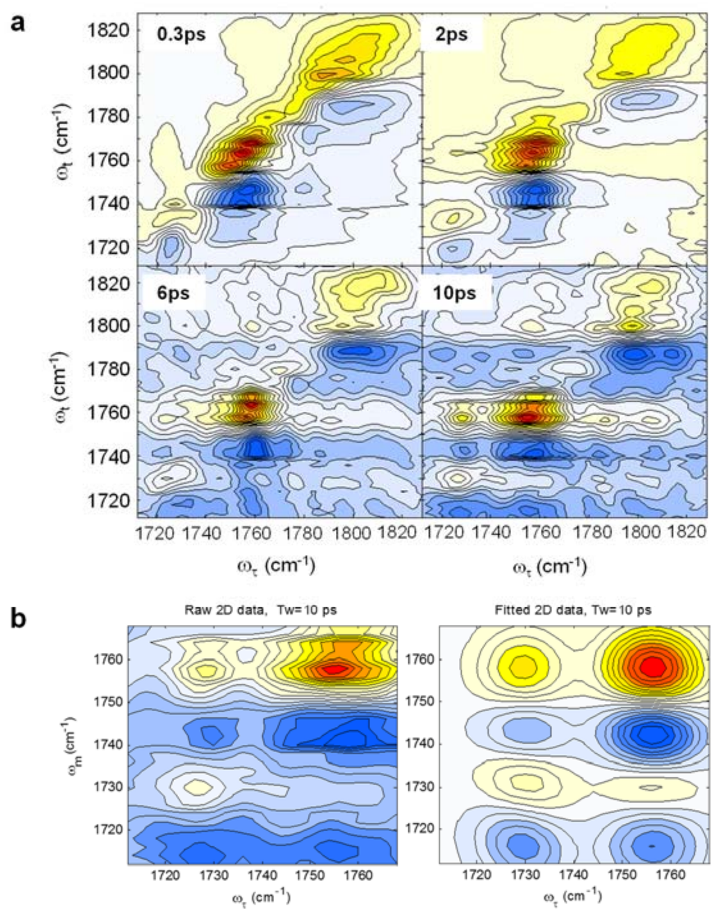

Supplementary Figure 11. Time-resolved 2DIR chemical exchange spectra for the solution (ii), 1.5 M $LiPF_6$ in DMC:PC (= 1.5:1) solution.

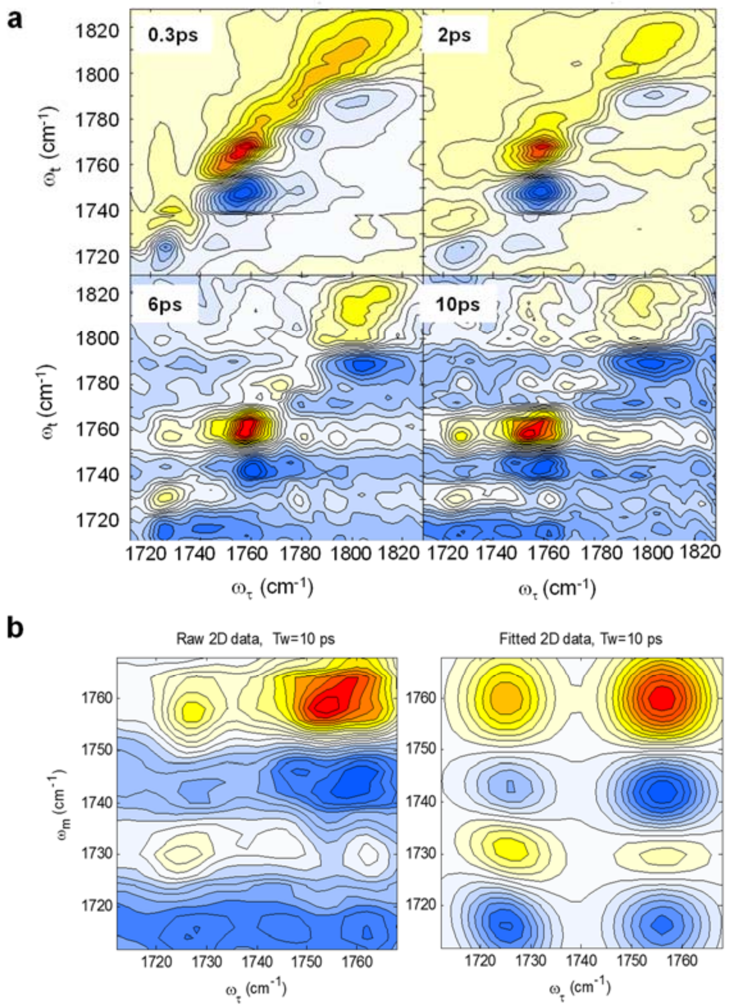

Supplementary Figure 12. Time-resolved 2DIR chemical exchange spectra for the solution (iii), 2.0 M $LiPF_6$ in DMC:PC (= 1.5:1) solution.

Supplementary Table 1. Density functional theory (B3LYP/6-311++G(3df,2pd)) calculation (both geometry optimization and vibrational analysis) results of the two lithium DEC complex forms in Fig. 2. Frequency shifts of C=O stretch and O-C-O asymmetric stretch modes of the two complex forms were calculated by comparing them with those of an isolated (gas-phase) DEC molecule. The bond length changes upon complex formation are presented here.

DEC species	C=O···Li^+ (Fig. 2**a**)	O=C-O···Li^+ (Fig. 2**b**)
$\Delta\omega$ (cm^{-1}) of C=O stretch	-112.2	112.4
$\Delta\omega$ (cm^{-1}) of O-C-O asymmetric stretch	62.6	-96.8
ΔR (Å) of C=O bond length	0.037	-0.023
ΔR (Å) of C-O bond length	-0.035	0.024

Supplementary Table 2. The vibrational frequencies and transition dipole moments calculated with DFT (B3LYP/6-311++G(3df,2pd)) are presented for $Li^+(DEC)_4$ and $(DEC)_3Li^+:PF_6^-$ complexes. The transition dipole moments are in Debye per Å. In the case of an isolated DEC molecule, the vibrational frequency and transition dipole moment of C=O stretch mode are 9.669 D/Å. The number inside parenthesis is the ratio of transition dipole moment of DEC species considered here to that of an isolated DEC.

DEC species	ω (cm^{-1}) of C=O stretch	Transition dipole (D/Å) of C=O stretching
Li^+[CCCC]	1664.9	15.5 (1.6)
[CCC] $Li^+:PF_6^-$	1670.2	15.0 (1.6)
Li^+[CCCT]	1660.4	14.3 (1.5)
[CCT] $Li^+:PF_6^-$	1674	17.6 (1.8)
Li^+[CCTT]	1681.8	19.7 (2.0)
[CTT] $Li^+:PF_6^-$	1680.2	16.5 (1.7)
Li^+[CTTT]	1687.63	19.4 (2.0)
[TTT] $Li^+:PF_6^-$	1704.4	19.5 (2.0)
Li^+[TTTT]	1689.0	18.1 (1.9)

Supplementary Note 1.
Radio-frequency magnetron sputtering method

For the FT-IR, IR pump-probe, and 2DIR measurements, the IR absorbance value of the sample should be in a proper range. If the absorbance is too high, the absorption spectrum is saturated and the nonlinear IR signal is re-absorbed by the sample so that it is difficult to measure the third-order nonlinear IR signals. If the absorbance is too low, obviously it is difficult to measure the signal against noise. Therefore, the amount of sample in IR cell needs to be controlled, which was usually achieved by changing the thickness of sample cell with various Teflon spacers. However, in the present work, the solvent DEC itself is the IR probe. Therefore, we needed to have a very thin sample cell. Unfortunately, there is no Teflon spacer with sub-micrometer thickness. Furthermore, we wanted to build an IR sample cell that is suitable for the cell holder in commercial FT-IR spectrometer. We thus found that sputter deposition, which is a physical vapor deposition method of thin film deposition by sputtering, is an ideal approach. This involves ejecting material from a "target" that is chosen to be SiO_2 onto a "substrate" that is CaF_2 window.

We used a radio frequency (RF) magnetron sputtering system. The sputtering source employs magnetrons that utilize strong electric and magnetic fields to confine charged plasma particles close to the surface of the sputter target SiO_2. The sputter gas used is argon. Note that the extra argon ions created through collisions with electrons lead to a higher deposition rate. The sputtered atoms are neutrally charged so that they are not affected by the magnetic trap. To avoid any charge build-up on the insulating SiO_2 target, the sign of the anode-cathode bias is varied at a high rate that is in the radio frequency of 13.56 MHz. It has been known that RF magnetron sputtering works very nicely to produce highly insulating oxide films[1].

Supplementary Fig. 1 shows a schematic representation of RF magnetron sputtering system used to prepare a home-built IR sample cell with ring-shape thin SiO_2 film on CaF_2 window. The target for the growth of SiO_2 film on a substrate was 2-inch disk of sintered SiO_2 (99.999 %, 1/8 inch thick, TASCO Inc.) with a copper back plate (2 inch diameter, 3 mm thick). CaF_2 window should be carefully chosen so that its surface is sufficiently flat as compared to the thickness (IR beam path length) of SiO_2 film. The CaF_2 windows (25.4 mm diameter, 3 mm thick), which have flatness below $\lambda/20$ at 633 nm, were especially purchased from Photop Tech. The CaF_2 substrate was then cleaned in the ultrasonic baths of acetone, ethanol, and deionized water for 10 minutes each, and dried with nitrogen gas. After putting the substrate into the sputtering chamber, the chamber was evacuated to a base pressure at 5.0

× 10^{-5} Pa for an hour. The working pressure was maintained at 1.30 Pa with high purity Ar (99.999%), which was used as the sputtering gas. The distance between the target and the substrate was 50 mm. Prior to the film deposition, the target was pre-sputtered for 20 min to remove any contaminant before opening the shutter covering the substrate. The film was sputtered at 25 °C with the RF power of 100 W. The film thickness was adjusted by controlling the operating time. As shown in Supplementary Fig. S1, a round-shaped polyimide film with diameter of 13 mm was used on the CaF_2 substrate during the sputtering, and then removed after completing the deposition of SiO_2.

We monitored the thickness of SiO_2 film on CaF_2 by using scanning electron microscopy (SEM) and found a linear relationship between the sputtering time and the thickness of the SiO_2 film (Fig. 1**b**). Therefore, the thickness of the layer, which is approximately identical to the IR beam path length, could be easily controlled. We found that ~800 nm beam path length is appropriate for the present FT-IR and time-resolved nonlinear IR spectroscopic investigations of $LiPF_6$ DEC solutions. Here, note that it is not absolutely necessary to control the thickness of SiO_2 layer accurately, as long as the IR absorbance of solution sample is about 0.3, which is an optimum value for the present IR pump-probe and 2DIR measurements.

Supplementary Note 2.

FT-IR spectroscopy and quantum chemistry calculations.

Assignment of a newly emerging band at 1715.4 cm^{-1}. In the main text, we assigned the absorption band at 1715.4 cm^{-1}, which emerges as the lithium salt concentration is increased, to the C=O stretch mode of DEC when its carbonyl oxygen atom makes a strong electrostatic interaction with Li^+. To obtain further evidence on this assignment, we carried out density functional theory (DFT) calculations with the Gaussian09[2] program at the level of B3LYP/6-311++G(3df,2pd). We could deliberately change the distance between Li^+ and the carbonyl oxygen atom from 1.76 to 10 Å (Supplementary Fig. 2). At each fixed distance R, the molecular structure of DEC was geometry-optimized and the vibrational frequencies were obtained. As shown in figure, the C=O stretch mode frequency (red circles) decreases as the lithium cation approaches to the carbonyl oxygen atom, whereas the O-C-O asymmetric stretch mode frequency (blue circles) undergoes the opposite frequency shift. In particular, the frequency shifts of the two modes, i.e., C=O stretch and O-C-O asymmetric stretch, are large when Li^+ is close to the carbonyl oxygen atom in less than 3 to 4 Å. The new peak at 1715.4 cm^{-1} in Fig. 1**c** indicates that the lithium binding site is the carbonyl oxygen atom of DEC not the ester ether oxygen atoms and that the lithium ions beyond the first solvation shell do not induce much frequency shifts of the two modes.

The carbonyl IR stretch bands associated with those of C=O···Li^+ and C=O···Li^+:PF_6^- complexes cannot be spectrally resolved. Quantum calculation results show that the carbonyl stretch IR band of C=O···Li^+:PF_6^- complexes will appear at frequency region between those of pure DEC and (DEC)C=O···Li^+ complex. To investigate a spectrally discernible (low-frequency) feature associated with the C=O···Li^+:PF_6^- complex, we compare the normalized C=O band of $LiPF_6$ DEC solution with the normalized C=O spectrum of pure DEC (Supplementary Fig. 3). After subtracting the pure DEC spectrum from the normalized C=O stretch IR spectra of $LiPF_6$ DEC solutions, we obtained the difference spectra (red lines) and found no notable (spectrally distinguishable) low-frequency peak other than the main new peak at 1715.4 cm^{-1}. In the difference spectra (red), the line shape at around the main (free DEC) peak at ~1750 cm^{-1} appears to be dispersive with positive and negative feature. This is due to the chemical exchange-induced line broadening of the C=O stretch IR spectrum of free DEC molecules in the solution[3]. Note that such additional exchange dynamics between free (unbound) DEC and bound (lithium-complexed) DEC is absent in pure DEC

liquid. Overall, the FTIR spectral analysis in figure clearly shows that the C=O···Li^+ complex and the C=O···Li^+:PF_6^- complex cannot be spectrally resolved and that they both contribute to the new peak at 1715.4 cm^{-1} in the FTIR spectra of $LiPF_6$ DEC solutions.

Li-DEC complex structure. In the main text, we considered three Li-DEC complex structures that are C=O···Li^+, C=O···Li^+:PF_6^-, and O=C(-O)$_2$:::Li^+. Here, the first and third cases (C=O···Li^+ and O=C-O···Li^+) represent the Li-DEC complexes with Li^+ surrounded by other DEC molecules. In contrast, the second case (C=O···Li^+:PF_6^-) corresponds to the DEC molecule interacting with Li^+:PF_6^- (contact ion pair). From the FT-IR data, NMR results, and our quantum chemistry calculation results, we ruled out the possibility that Li^+ forms a strong electrostatic interaction with ester ether oxygen atoms. However, there is yet another solvation structure that involves the Li^+ ion in CIP (Li^+:PF_6^-) interacting with the two ester ether oxygen atoms, i.e., O=C(-O)$_2$:::Li^+:PF_6^-. However, this possibility can also be easily ruled out because the corresponding frequency shifts of C=O stretch and O–C–O asymmetric stretch modes, which are predicted by our DFT calculation of the O=C(-O)$_2$:::Li^+:PF_6^- complex, are not consistent with the present experimental results shown in Fig. 1. Therefore, from the present quantum chemistry calculations (Supplementary Fig. 2) and FTIR spectral analysis (Supplementary Fig. 3), it is concluded that the new C=O stretch IR absorption peak appearing at 1715.4 cm^{-1}, as $LiPF_6$ concentration increases, originates from the contributions from the C=O stretching vibrations of both C=O···Li^+ and C=O···Li^+:PF_6^- complexes. This peak was thus referred to as the Li-DEC complex peak in the main text.

Quantum chemistry calculation for contact ion pair and free ion. As shown in the IR spectrum in Fig. 1**c**, there are two peaks though there could exist three different DEC species that are DEC in C=O···Li^+ complex, DEC in C=O···Li^+:PF_6^- complex, and free DEC. Our FTIR analysis results (Supplementary Fig. 2) show that the peak at 1715.4 cm^{-1} originates from both DEC species in C=O···Li^+ and C=O···Li^+:PF_6^- complexes. If one further considers various conformations of the ethyl ether group of each DEC molecule, there could be many more conformationally different Li-DEC complexes. However, such minor differences in the side chain conformation do not make any IR spectroscopically distinguishable difference in the spectrum as shown in Supplementary Fig. 3.

Our quantum chemistry calculations have thus been used to interpret the C=O stretch IR absorption spectra of Li-DEC solutions. In the previous literatures, the solvation number of DEC molecules were estimated to be about 3 to 4, by using the concentration dependent IR and/or Raman spectroscopic measurements. In addition, resent quantum chemistry

calculation studies showed that the average solvation number is about 3 to 4. If one PF_6^- ion participates in the solvation as a ligand replacing DEC, two to three DEC molecules participate in solvating Li^+. In that case, the effective number of DEC molecules in the first solvation shell decreases due to the extra involvement of PF_6^- in the solvation, which makes the absorption intensity at 1715.4 cm^{-1} smaller than that of the case when Li^+ is fully solvated by DEC molecules only. Therefore, we carried out additional DFT calculations for $Li^+(DEC)_4$ and $(DEC)_3Li^+:PF_6^-$ with a variety of possible ethyl group conformations. Here, we use abbreviated notations for different conformers of DEC as C and T, which represent *cis-cis* and *cis-trans* ethyl conformation, respectively. Conformer with *trans-trans* configuration is not populated because its energy is much higher than others due to the steric repulsion between the two ethyl groups. For $Li^+(DEC)_4$, we need to consider five conformers like Li^+[CCCC], Li^+[CCCT], Li^+[CCTT], Li^+[CTTT], and Li^+[TTTT]. The quantum chemistry optimization structures are shown in Supplementary Fig. 4**a**. Their vibrational frequencies and transition moments are summarized in Supplementary Table 2. Due to vibrational couplings among C=O stretch modes, the numerically simulated (with Lorentzian lineshape functions) exhibit four bands (Supplementary Fig. 4**b**).

Among them, we consider the band with the largest transition dipole moment and the corresponding frequencies are presented in Supplementary Table 2. In the case of $(DEC)_3Li^+:PF_6^-$, four different conformers were considered for the present quantum chemistry geometry optimization, which are [CCC] $Li^+:PF_6^-$, [CCT] $Li^+:PF_6^-$, [CTT] $Li^+:PF_6^-$, and [TTT] $Li^+:PF_6^-$. Comparing these computation results, we found that the C=O stretch frequencies of $(DEC)_3Li^+:PF_6^-$ complexes are slightly larger than those of $Li^+(DEC)_4$ (see Supplementary Fig. 4**b**). Another observation is that the transition dipole moments of the structures shown in Supplementary Fig. 4**a** are in the range from 14.3 and 19.5 D/Å. They are, on average, larger by 1.7 than that of free DEC without making any electrostatic interaction with Li^+. This value of 1.7 is in excellent agreement with that estimated from experimental results.

Supplementary Note 3.

IR pump-probe data and analysis results

For polarization-controlled pump-probe experiments, the pump and probe pulses were linearly polarized with a wire grid polarizer. Before the sample stage, the probe polarization was set to be 45° with respect to the pump polarization direction. After the probe pulse interacts with the sample, it passes through a motorized polarizer and the transient probe signals with polarizations of both 0° and 90° with respect to the pump polarization are measured with monochromator and MCT (Mercury Cadmium Telluride) array detector. The parallel $I_{\parallel}(\omega_{pr},t)$ and perpendicular $I_{\perp}(\omega_{pr},t)$ pump-probe signals detected are used to obtain the isotropic (eq.(1)) and anisotropic (eq.(2)) signals that are defined as

$$P_{\text{iso}}(t) = \frac{I_{\parallel}(t) + 2I_{\perp}(t)}{3} \text{ and} \quad (1)$$

$$R(t) = \frac{I_{\parallel}(t) - I_{\perp}(t)}{I_{\parallel}(t) + 2I_{\perp}(t)}. \quad (2)$$

Frequency-dependent decay constant of isotropic PP signal. Supplementary Fig. 5**(top)** show the vibrational lifetime analysis results for pure DEC liquid and $LiPF_6$-DEC solution, respectively. The isotropic pump-probe spectra at waiting time of 0.3 ps are shown in these two panels. Furthermore, we fit the time-dependent isotropic pump-probe signal at each probe frequency with a single exponential function. The fitted decay constant is the vibrational lifetime at each probe frequency. The fit constants (lifetimes) are also plotted (see red squares). We intentionally avoid analyzing the isotropic signals in the probe frequencies where the positive 0-1 peak overlaps with the negative 1-2 peak. The frequency-dependent decay constant obtained by analyzing the time-dependent decay of the 0-1 positive signal increases with probe frequency. This is mainly due to the undesired contribution from local heating induced by pump. Interestingly, the vibrational lifetimes extracted from the analyses of the negative 1-2 peak are not strongly dependent on the probe frequency. This is mainly because the negative 1-2 peak is not strongly affected by the same local heating effect in these cases. Typical fitting results are shown in Supplementary Fig. 5**(bottom)**, which show that the isotropic PP signal from pure DEC fits well with a single exponential function. Despite that a bi-exponential fitting analysis of the isotropic pump-probe signals of $LiPF_6$-DEC solution works slightly better than that with a single exponential function, the fit quality

with the latter is still excellent so that we report and use this single exponential fit result on the C=O stretch vibrational lifetime of the Li-DEC complex.

Lithium salt concentration-dependence of C=O stretch vibrational lifetime of free DEC. Since vibrationally excited DEC molecule is always surrounded by other DEC molecules in solution, its energy can be transferred to the other ground-state DEC molecules via resonant energy transfer mechanism. Often, if solvent molecules are chosen to be IR probes, e.g., O-H stretch mode of water in aqueous solution, to avoid any complexation caused by resonant excitation transfers among IR probes, an isotope dilution method has been used because the isotope labeled probe, e.g., O-D stretch mode of HOD in aqueous water, is spectrally separated from those of surrounding solvent molecules[4]. Since DEC molecules in our sample solutions, which are very close to realistic electrolyte solutions used in LIB, are not only IR probes but also solvent, we should carefully examine the possibility of the 2DIR cross peaks that can be produced by vibrational excitation transfer between two C=O stretch modes of free DEC and Li-DEC complex. To address this problem, we carried out a series of concentration-dependent pump-probe measurements at four different concentrations of $LiPF_6$ from 0.2 to 1.0 M. The decrease in $LiPF_6$ concentration would significantly reduce the chance for free DEC to encounter Li-complexed DEC molecules. This should increase the vibrational lifetime of free DEC, if the vibrational energy transfer between free DEC and DEC in Li-DEC complex occurs within a few picosecond time scale. However, as shown in Supplementary Fig. 6, the vibrational lifetime of free DEC is almost independent of $LiPF_6$ concentration.

More specifically, as $LiPF_6$ concentration changes, vibrational lifetimes do not change much and are in the range from 2.2 to 2.4 ps. This indicates that the cross peaks observed in our 2DIR spectra originate from ultrafast dynamic equilibrium and solvent chemical exchange process in and out of the Li-ion solvation sheath in LIB electrolyte solution, not from simple vibrational excitation transfer between free DEC and Li-DEC complex.

Vibrational energy relaxation mechanism. Vibrational energy relaxation (VER) rate of the C=O stretch mode of DEC increases upon its complex formation with Li^+. The vibrational lifetime of C=O stretch in pure DEC liquid is 2.1 ps, whereas that of Li-DEC complex is 1.1 ps. Since the frequency of the $O\cdots Li^+$ stretch mode is much different from the C=O stretch frequency, the vibrational energy relaxation of C=O stretch mode to low-frequency $O\cdots Li^+$ stretch mode is not efficient. Therefore, the intermolecular vibration of Li-DEC complex may not be responsible for the increased VER rate upon Li-complex formation of DEC. Even in

pure DEC liquid, the C=O oscillator shows a very rapid VER. Therefore, the VER of C=O stretch mode of free DEC and Li-DEC complex should involve intramolecular VER processes. For an efficient vibrational excitation transfer, the energy difference between donor and acceptor states is better to be small. In addition, vibrational anharmonic couplings are prerequisite for such fast intramolecular VER[5]. In the case of free DEC, the O−C−O asymmetric stretch band is close in frequency to the C=O stretch band and furthermore these vibrational motions involve the movement of a common atom that is the carbonyl carbon. Thus, the O−C−O asymmetric (and/or symmetric) stretching mode can be the primary accepting mode in the intramolecular VER process of the C=O stretch. Now, as DEC forms a complex with Li^+, the C=O stretch frequency is red-shifted and the O-C-O asymmetric stretch frequency is blue-shifted. Consequently, the energy gap between the donor and acceptor modes in the case of the Li-DEC complex becomes less than 100 cm^{-1}. This facilitates the increase of intramolecular VER rate of C=O stretch excitation when DEC forms a complex with Li^+.

However, there is another possible relaxation pathway that needs to be considered. Since vibrationally excited DEC molecules are surrounded by other DEC molecules, the excited C=O stretch mode can transfers its vibrational energy to neighboring molecules. However, we can rule out the possibility that resonant energy transfer among DEC molecules contributes to the VER. If the intermolecular resonant energy transfer is the dominant VER pathway, any further addition of $LiPF_6$ to the DEC solution would slow down the intermolecular vibrational excitation transfer. This is because the increased concentrations of Li^+ and PF_6^- effectively dilute DEC solution, which causes an increase of mean intermolecular distance between nearest neighboring DEC molecules. However, this is in contradiction with our experimental finding that the C=O vibrational lifetime decreases as lithium salt concentration increases. Therefore, the fact that the vibrational lifetime of Li-DEC complex (1.1 ps) is much faster than that of free DEC led us to conclude that the intermolecular resonant energy transfer does not contribute to the fast VER of the C=O stretch of DEC molecules in Li-DEC complex.

Supplementary Note 4.

Ratio of the transition dipole strength of Li-DEC to that of free DEC

Concentration-dependence of the C=O stretch IR spectrum can be used to determine the ratio of the transition dipole moment of C=O stretch mode of free (unbound) DEC to that of Li-DEC complex. The integrated area of the C=O stretch IR band at 1747 cm^{-1}, which is denoted as I_f (free DEC), and that at 1715.4 cm^{-1}, denoted as I_c (Li-DEC), can be obtained from fitting analysis of each spectrum in Fig. 1**c** with two Gaussian functions. The total integrated areas at various $LiPF_6$ concentrations are also plotted in Supplementary Fig. 8**a**. It is noted that the total intensity (black triangles) increases with respect to increasing $LiPF_6$ salt concentration. In fact, as the salt concentration increases, the effective number of DEC molecules in the volume determined by the IR beam diameter (or spot size) and beam path length should decrease. This would lead to a decrease of the total absorbance, if the transition dipole moment of Li-DEC complex is quantitatively similar to that of free DEC. However, the observation that the total intensity I_t increases with respect to the increase in $LiPF_6$ concentration clearly indicates that the transition dipole moment of Li-DEC is significantly larger than that of free DEC.

Unfortunately, it is not easy to determine the ratio of the transition dipole moment of free DEC to that of Li-DEC complex with one FTIR spectrum only, because the relative population ratio between complex and free DEC is not known. However, it is possible to use a series of concentration-dependent FTIR spectra. Before that, it should be noted that, from our quantum chemistry calculation results for those shown in Supplementary Fig. 4, the transition dipole moment of C=O stretch mode of DEC$\cdots$$Li^+$ is almost the same with that of DEC$\cdots$$Li^+$:$PF_6^-$. Therefore, the Li-DEC complex refers to both DEC molecules in DEC$\cdots$$Li^+$ and DEC$\cdots$$Li^+$:$PF_6^-$. Then, the total area can be expressed with transition dipole and concentrations of free DEC (C_f) and Li-DEC complex (C_c) as

$$I_t = I_f + I_c = C_f \mu_f^2 + C_c \mu_c^2 = \left(1 - \mu_f^2 / \mu_c^2\right) I_c + \mu_f^2 C_t = \left(1 - \mu_c^2 / \mu_f^2\right) I_f + \mu_c^2 C_t \quad (3)$$

where C_t is the total concentration of DEC. Here, C_t can be safely assumed to be constant in the concentration range of the present study. From eq.(3), we find a linear relationship between I_t and I_f or between I_c and I_f as shown in Supplementary Fig. 8**b**. From the slope of this linear fit, we found that the ratio of the transition dipole moment of Li-DEC to that of free DEC is 1.7.

Supplementary Note 5.

FTIR evidence supporting the participation of DEC in the solvation of Li^+ even in mixed solvent with propylene carbonate

As emphasized in the main text, there exist controversies under intense debates about the first solvation shell structure around Li-ion in mixed solvent system. The composition of the first solvation shell is extremely important in understanding the dynamics of Li^+ ion transport as well as its behaviors at the interface between electrolyte solution and solid electrodes. Recently, for a mixed solvent system with equal volumetric DEC and PC, Raman spectroscopy with density functional theory calculation was reported and showed that DEC molecules can also participate in the first solvation shell around Li^+, even though the solvating power of PC is higher than that of DEC. From these experimental results, they suggested that the primary solvation structure is $[(PC)_3(DEC)Li^+]$ with three PC and one DEC molecules[6]. In that work, the Raman spectra in the region at around 900 cm^{-1} revealed the direct interaction between C=O and Li^+. In the present FTIR measurements, it is clearly shown than the C=O stretching vibration band from Li-DEC complex is frequency-resolved from that of free DEC molecules. Due to the increase of C=O stretch transition dipole moment when DEC's C=O forms an electrostatic interaction with Li^+, the Li-DEC complex formation can be easily detectable with FTIR measurement of the C=O stretch band.

To further confirm the participation of co-solvent DEC or DMC molecule in the lithium solvation sheaths even in mixed (either DEC:PC or DMC:PC) solvent systems, we measured the FTIR spectra of four mixed-solvent solutions: (i) 1.0 M $LiPF_6$ in DMC:PC (=1:1 in volume percent) solution, (ii) 1.5 M $LiPF_6$ in DMC:PC (= 1.5:1) solution, (iii) 2.0 M $LiPF_6$ in DMC:PC (= 1.5:1) solution, and (iv) 1.0 M $LiPF_6$ in DEC:PC (=1:1) solution (Supplementary Fig. 9).

In Supplementary Fig. 9, the carbonyl (C=O) stretch IR bands of the four solutions mentioned above are plotted. In Supplementary Fig. 9**b**, the C=O stretch IR bands of two different solutions, which are 1.0 M $LiPF_6$ DEC solution (red line) and 1.0 M $LiPF_6$ PC solution (blue line), are also shown for the sake of direct comparison. The C=O stretch band of free PC is at around 1800 cm^{-1}, whereas that of Li-PC complex appears at around 1775-1780 cm^{-1}. For instance, the carbonyl stretch IR spectrum of 1.0 M $LiPF_6$/DEC:PC (1:1 in volume percent) solution in Supplementary Fig. 9**d** shows four discernable bands. If Li^+ in this mixed solvent solution is completely solvated by PC not DEC, there must not be a band

at ~1715 cm^{-1}, which is the C=O stretch band of Li-DEC complex. However, the clear appearance of 1715 cm^{-1} peak in the IR spectrum (black line) of 1.0 M $LiPF_6$/DEC:PC (1:1 in volume percent) solution confirms that DEC molecules can also participate in solvating Li^+ ion too, which further suggests that co-solvent, either DMC or DEC, with comparatively low viscosity acts as not just a medium for transport of lithium solvation sheath but also a ligand directly making Coulombic interaction with Li^+. As can be seen in Supplementary Fig. 9**a** for the solutions (i)-(iii) mentioned above, there is a clear spectroscopic feature at around 1725 cm^{-1} indicating the existence of Li-DMC complex. This also manifests that the low viscous DMC solvent compared to PC does participate in forming the first solvation shell around Li^+.

Supplementary Note 6.
2DIR chemical exchange spectroscopy of mixed solvent systems

We carried out 2DIR chemical exchange spectroscopic studies of three solutions mention in section VI above. The time-resolved 2DIR spectra are shown in Supplementary Fig. 10-12. Due to the presence of diagonal and cross peaks originating from Li-PC complexes and free PC molecules, the experimentally measured 2DIR spectra appear to be highly congested. Nonetheless, one can easily observe the increase of the cross peak intensity between Li-DMC and free DMC in the latter two solutions (ii) and (iii). We further performed two-dimensional Gaussian fittings to the experimental spectra in Supplementary Fig. 11 and 12, which allowed us to estimate the ratio of the cross peak intensity to the square root of the product of the two diagonal peaks, i.e., $I^{\mathbf{cross}}_{\mathbf{Li\text{-}DMC:DMC}} / \sqrt{I^{\mathbf{diag}}_{\mathbf{Li\text{-}DMC}} I^{\mathbf{diag}}_{\mathbf{DMC}}}$.

Supplementary references

1 Sun, H.-J. Thermal Instability of $La_{0.6}Sr_{0.4}MnO_3$ Thin Films on Fused Silica. *Korean J. Mater. Res.* **21**, 482-485 (2011).

2 Frisch, M. J. *et al*. *Gaussian 09, Revision C.01* (Gaussian, Inc., Wallingford, CT, USA, 2009).

3 Kwak, K., Zheng, J., Cang, H. & Fayer, M. D. Ultrafast two-dimensional infrared vibrational echo chemical exchange experiments and theory. *J. Phys. Chem. B* **110**, 19998-20013 (2006).

4 Cho, M. Coherent two-dimensional optical spectroscopy. *Chem. Rev.* **108**, 1331-1418 (2008).

5 Hamm, P., Lim, M. H. & Hochstrasser, R. M. Structure of the amide I band of peptides measured by femtosecond nonlinear-infrared spectroscopy. *J. Phys. Chem. B* **102**, 6123-6138 (1998).

6 Giorgini, M. G., Futamatagawa, K., Torii, H., Musso, M. & Cerini, S. Salvation Structure around the Li^+ Ion in Mixed Cyclic/Linear Carbonate Solutions Unveiled by the Raman Noncoincidence Effect. *J. Phys. Chem. Lett.* **6**, 3296-3302 (2015).
